# Gut dysbiosis in patients with chronic pain: a systematic review and meta-analysis

**DOI:** 10.3389/fimmu.2024.1342833

**Published:** 2024-01-30

**Authors:** Lisa Goudman, Thomas Demuyser, Julie G. Pilitsis, Maxime Billot, Manuel Roulaud, Philippe Rigoard, Maarten Moens

**Affiliations:** ^1^ STIMULUS (Research and Teaching Neuromodulation Uz Brussel) Research Group, Vrije Universiteit Brussel, Brussels, Belgium; ^2^ Department of Neurosurgery, Universitair Ziekenhuis Brussel, Brussels, Belgium; ^3^ Center for Neurosciences (C4N), Vrije Universiteit Brussel, Brussels, Belgium; ^4^ Pain in Motion (PAIN) Research Group, Department of Physiotherapy, Human Physiology and Anatomy, Faculty of Physical Education and Physiotherapy, Vrije Universiteit Brussel, Brussels, Belgium; ^5^ Research Foundation—Flanders (FWO), Brussels, Belgium; ^6^ Charles E. Schmidt College of Medicine, Florida Atlantic University, Boca Raton, FL, United States; ^7^ Department Microbiology and Infection Control, Universitair Ziekenhuis Brussel, Brussels, Belgium; ^8^ AIMS Lab, Center for Neurosciences, Faculty of Medicine and Pharmacy, Vrije Universiteit Brussel, Brussels, Belgium; ^9^ PRISMATICS Lab (Predictive Research in Spine/Neuromodulation Management and Thoracic Innovation/Cardiac Surgery), Poitiers University Hospital, Poitiers, France; ^10^ Department of Spine Surgery and Neuromodulation, Poitiers University Hospital, Poitiers, France; ^11^ Pprime Institute UPR 3346, CNRS, ISAE-ENSMA, University of Poitiers, Chasseneuil-du-Poitou, France; ^12^ Department of Radiology, Universitair Ziekenhuis Brussel, Brussels, Belgium

**Keywords:** microbiota, gut-brain axis, persistent pain, biomarker, gut composition, stool samples

## Abstract

**Introduction:**

Recent evidence supports the contribution of gut microbiota dysbiosis to the pathophysiology of rheumatic diseases, neuropathic pain, and neurodegenerative disorders. The bidirectional gut-brain communication network and the occurrence of chronic pain both involve contributions of the autonomic nervous system and the hypothalamic pituitary adrenal axis. Nevertheless, the current understanding of the association between gut microbiota and chronic pain is still not clear. Therefore, the aim of this study is to systematically evaluate the existing knowledge about gut microbiota alterations in chronic pain conditions.

**Methods:**

Four databases were consulted for this systematic literature review: PubMed, Web of Science, Scopus, and Embase. The Newcastle-Ottawa Scale was used to assess the risk of bias. The study protocol was prospectively registered at the International prospective register of systematic reviews (PROSPERO, CRD42023430115). Alpha-diversity, β-diversity, and relative abundance at different taxonomic levels were summarized qualitatively, and quantitatively if possible.

**Results:**

The initial database search identified a total of 3544 unique studies, of which 21 studies were eventually included in the systematic review and 11 in the meta-analysis. Decreases in alpha-diversity were revealed in chronic pain patients compared to controls for several metrics: observed species (SMD= -0.201, 95% CI from -0.04 to -0.36, p=0.01), Shannon index (SMD= -0.27, 95% CI from -0.11 to -0.43, p<0.001), and faith phylogenetic diversity (SMD -0.35, 95% CI from -0.08 to -0.61, p=0.01). Inconsistent results were revealed for beta-diversity. A decrease in the relative abundance of the Lachnospiraceae family, genus *Faecalibacterium* and *Roseburia*, and species of *Faecalibacterium prausnitzii* and *Odoribacter splanchnicus*, as well as an increase in *Eggerthella* spp., was revealed in chronic pain patients compared to controls.

**Discussion:**

Indications for gut microbiota dysbiosis were revealed in chronic pain patients, with non-specific disease alterations of microbes.

**Systematic review registration:**

https://www.crd.york.ac.uk/prospero/, identifier CRD42023430115.

## 1 Introduction

The gut microbiota refers to the dynamic community of microorganisms inhabiting the gastro-intestinal tract, whereby the genetic and functional profile of microbial species is denoted as the gut microbiome (1, 2). During the last decade, several studies pointed out associations between alterations in microbiota composition and diverse host disease conditions, among those gastrointestinal conditions [e.g., irritable bowel syndrome (3), gastroduodenal diseases (4)] as well as more physically remote conditions among which neurodegenerative diseases (e.g., Parkinson’s disease, Alzheimer’s disease, or multiple sclerosis) (5), or neuropsychiatric disorders (6). To accomplish these complex involvements, neuro-immune-endocrine mediators underlie the bidirectional communication network between the gut and the central nervous system, i.e. the gut-brain axis (7). As such, the gut-brain crosstalk ensures the proper maintenance of gastrointestinal homeostasis, while it also connects the emotional and cognitive centers of the brain with peripheral intestinal functions and mechanisms through immune activation, intestinal permeability, and entero-endocrine signaling (8).

The hypothalamic pituitary adrenal (HPA) axis, as part of the limbic system, is the core stress efferent axis that reacts with secretion of corticotropin-releasing factor from the hypothalamus in response to stressors of any kind (e.g., emotion or stress), consecutively leading to adrenocorticotropic hormone secretion from the pituitary gland, which in turn leads to cortisol release from the adrenal glands (9). While chronically elevated cortisol levels negatively affect brain function (10), HPA axis activation also alters the composition of the gut microbiota and increases gastrointestinal permeability (11), triggering an inflammatory response (12). Additionally, the autonomic nervous system drives both efferent signals from the central nervous system to the intestinal wall, mainly through vagal efferent fibers, and afferent signals from the lumen through enteric, spinal, and vagal pathways to the central nervous system (8). Unless the intestinal epithelium integrity is affected, whereby gut microbiota can directly interact with the vagal nerve, enteroendocrine cells recognize bacterial products or bacterial metabolites (e.g., short-chain fatty acids) to facilitate an indirect communication with vagal afferents through synaptic connections (13, 14). Additionally, production of bacterial metabolites (15), interference with the kynurenine pathway (16), and neuroendocrine signaling (17) contribute to the communication between the gut and the central nervous system.

Bidirectional interactions and connections between the pain regulatory system and the autonomic nervous system have been revealed (18), as well as altered sensitivity of the HPA axis in relation to chronic pain and stress (19), which are both suggestive of the involvement of the gut-brain axis in chronic pain due to shared pathways. Therefore, the aim of this study is to systematically evaluate the existing knowledge about gut microbiota alterations across a spectrum of chronic pain conditions.

## 2 Methods

### 2.1 Protocol registration

This systematic review was conducted according to the PRISMA statement (Preferred Reporting Items for Systematic Review and Meta-Analyses) (20). The protocol was *a priori* registered in PROSPERO under registration number CRD42023430115.

### 2.2 Search strategy

The search strategy was conducted in four databases: PubMed, Web of Science, Embase, and Scopus on June 3^rd^, 2023. All authors contributed to the development of the search strategy. The research question was created according to the PICOS (Population-Intervention-Control-Outcome-Study design) framework (21) to investigate perturbations in gut microbiota (Outcome) in chronic pain patients (Population). The final search strategy was built by combining both free and MeSH terms. Between each part of the PICO question, the Boolean operator AND was used. Within the components, search terms were combined using the Boolean operator OR. No limits were applied to this search strategy. The complete search strategy for PubMed can be found in **Supplementary Datasheet 1**. After building the search string in PubMed, it was individually adapted for the other three databases.

### 2.3 Eligibility criteria

Studies evaluating gut microbiota in chronic pain patients, in comparison to controls, were eligible. All types of chronic pain [pain > 3 months according to ICD-11 criteria (22)] were included, with the exception of functional intestinal disorders. As study designs, both observational and experimental designs were allowed, as long as a control group of patients without chronic pain was included. Only studies exploring gut microbiota were incorporated. Studies reporting in languages other than English, Dutch, or French were excluded. Full eligibility criteria are presented in **Table 1**.

**Table 1:** In-and exclusion criteria applied during screening for the systematic review.

*Topic	*Inclusion	*Exclusion
*Population*	- Chronic pain	- All types of chronic pain will be included, except for patients with functional intestinal disorders among which are irritable bowel syndrome, chronic ulceritis, functional abdominal pain, etc. - Animal studies, computational models
*Control*	- Healthy controls (defined as no presence of chronic pain)	
*Design*	- Observational designs (e.g. case-controls, cross-sectional, cohort designs) with cases and controls - Interventional or longitudinal comparisons with a control group.	- Reviews, case reports, letters to the editor, opinion articles, editorials - Interventional or longitudinal comparisons in the absence of a control group.
*Outcome*	- Measures of gut microbiota composition (alpha and beta diversity) and taxonomic findings at the phylum, family, and genus levels (relative abundance).	- Measures of the HPA axis, not related to the gut microbiome - Other microbiome than gut microbiome for example urinary microbiome or skin microbiome.
*Language*	- English, Dutch, French	- Other languages

HPA, hypothalamic pituitary adrenal.

### 2.4 Study selection

Two reviewers independently screened all retrieved articles for title and abstract using online software Rayyan, after de-duplication in both EndNote X9 and Rayyan. During the next phase, two reviewers independently performed full text screening. In case of conflicts at each stage, they were resolved in a consensus meeting with a third reviewer.

### 2.5 Data extraction

The relevant data were selected by an *a priori* developed data extraction form with information on publication details, participant demographic and clinical characteristics, and methodological information. As outcomes of interest, community-level measures of gut microbiota composition (alpha- and beta-diversity) and taxonomic findings at the phylum, family, genus, and species levels (relative abundance) were extracted. The alpha-diversity refers to the variation within an individual sample (i.e. microbial community) with a differentiation between richness (i.e. number of species) and evenness (i.e. how well each species is represented), while beta-diversity refers to the variation between samples (2, 23). The data extraction table was composed by one reviewer and checked for correctness by another reviewer. Any sort of discrepancies were discussed in a consensus meeting between both reviewers.

### 2.6 Quality assessment

The methodological quality of the included studies was evaluated with the Newcastle-Ottawa Scale (NOS), a tool developed for the purposes of evaluating nonrandomized studies used in systematic reviews and meta-analyses (24, 25). This scale is designed to assess the selection of participants (four items), comparability (one item), and exposure (three items) domains. A total NOS score ≤ 5 was considered as low quality, a score of 6 or 7 as moderate quality, and a score of 8 or 9 as high quality (26).

### 2.7 Data synthesis

Differences in alpha-diversity, beta-diversity, and relative abundance were qualitatively presented for patients with chronic pain, compared to controls. Additionally, random-effect meta-analyses were performed for alpha-diversity metrics (e.g. observed species, Chao1, abundance coverage estimator, Pielou, Shannon index, Simpson index, inverse Simpson index, and faith phylogenetic diversity) between chronic pain patients and controls in case ≥2 effect sizes were available for a specific metric. Standardized mean difference (SMD) was selected as metric for the meta-analyses, with the following interpretation: SMD ≤ 0.2 as trivial, < 0.2 < SMD < 0.5 as small, 0.5 ≤ SMD < 0.8 as moderate, and SMD ≥ 0.8 as large (23, 27). In case the necessary information could not be extracted adequately, the study authors were contacted to request it. When the median with the first and third quartile or interquartile range was provided, the mean and standard deviation were calculated manually, according to formulas provided by Wan et al. (2014) (28). In addition, if data were expressed only as a graph (rather than numerical data within the text), the software Engauge Digitizer 12.1 was used to extract numerical values. Heterogeneity was evaluated with I² statistic and publication bias with Egger’s test. All analyses were performed in R Studio version 2022.07.2. P values <0.05 were considered statistically significant.

## 3 Results

### 3.1 Study selection

**Figure 1:**
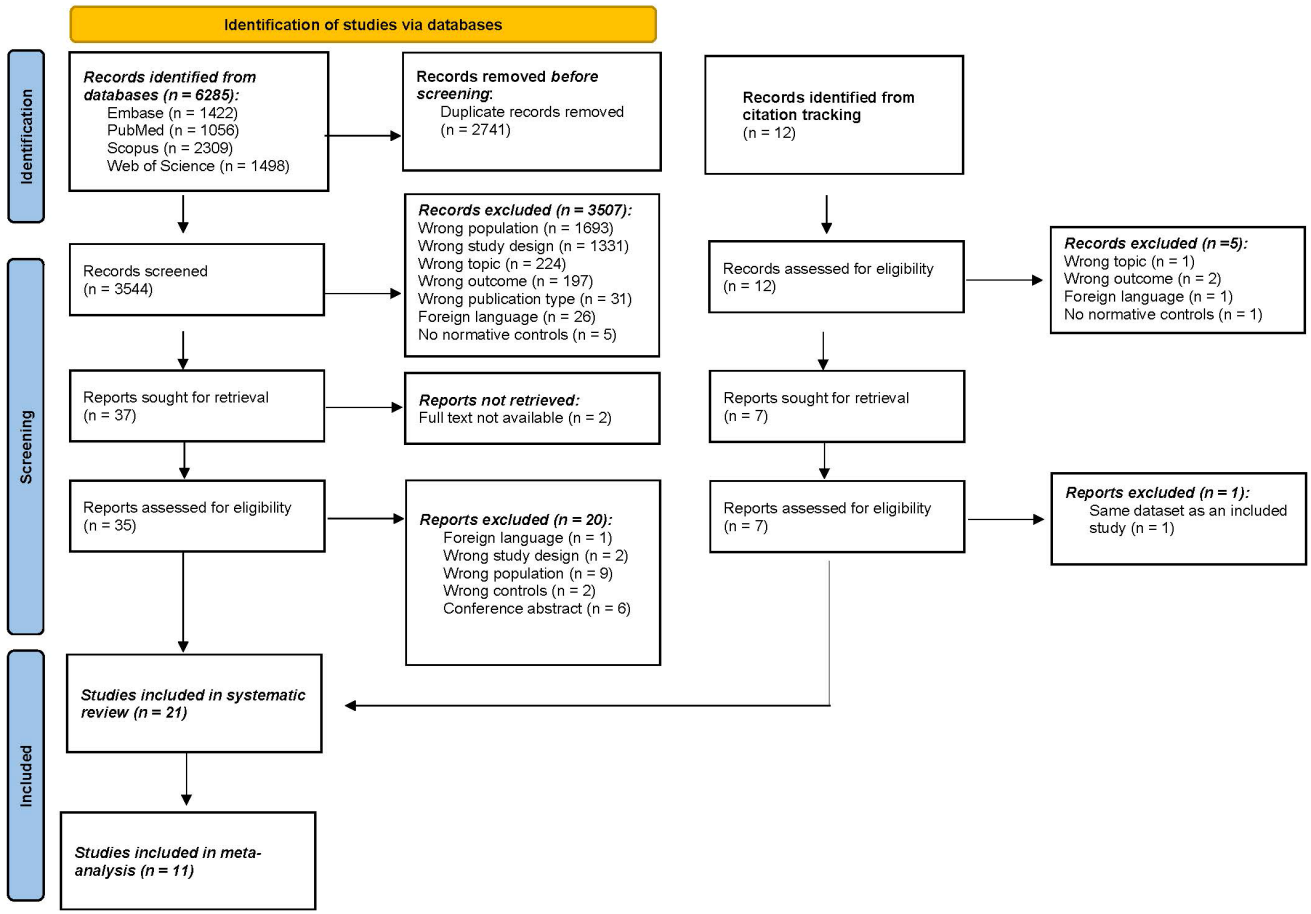
PRISMA (Preferred Reporting Items for Systematic Reviews and Meta-Analyses) flowchart. n, number.

A total of 6285 articles were identified through the four selected databases (**Figure 1**). After removing all duplicates, 3544 articles were selected for screening. After screening on title and abstract, 37 articles remained eligible for full screening. The percentage of agreement on title and abstract screening between both reviewers was 99.8% (7 conflicts). The reasons for exclusion were wrong population (n=1693), followed by wrong study design (n=1331), wrong topic (n=224), wrong outcome (n=197), and to a lesser extent wrong publication type, foreign language, and no controls. Afterward, 2 articles were excluded because there was no full text available. Citation screening identified 12 additional articles of which 7 were deemed suitable for full text screening. After full-text screening (N=42), 21 articles were included in this systematic review. The percentage of agreement on full text screening between both reviewers was 83.78%.

### 3.2 Study characteristics

Characteristics of the included studies are presented in **Table 2**. Nine studies (42.8%) were conducted in the USA, four (19%) in Asian countries, four (19%) in European countries, one (4.8%) in Canada, one (4.8%) in Australia, one (4.8%) in Ukraine, and one in the USA, UK, and Australia (4.8%). In terms of chronic pain populations, 19 studies explored chronic primary pain syndromes (pain is conceived as a disease), while 2 evaluated chronic secondary pain syndromes (pain manifests as a symptom of another disease). Specifically, 9 (42.8%) studies evaluated myalgic encephalomyelitis/chronic fatigue syndrome (ME/CFS), 4 (19%) studies included patients with migraine, and 3 (14.3%) studies evaluated patients with fibromyalgia. The following conditions were explored in only one study: axial spondyloarthritis (4.8%), interstitial cystitis/bladder pain syndrome (4.8%), Gulf War Illness (4.8%), complex regional pain syndrome (CRPS) (4.8%), and chronic stable angina (4.8%). In total, data from 962 chronic pain patients and data from 1212 controls without chronic pain were included. Patients and controls were matched in 9 studies on the following variables: age (9 studies), sex (7 studies), BMI (5 studies), geographical site/environment (3 studies), race/ethnicity (2 studies), date of sampling (1 study), season of sampling (1 study), and general activity patterns (1 study). The NOS of the included studies ranged from 2-9, with 10 studies classified as low quality, 4 as moderate quality, and 7 as high quality (**Supplementary Table 1**).

**Table 2:** Characteristics of the included studies.

Author	Country	Population	Sample size (with stool samples)	Age	Mean BMI	% Female	% Patients on medication	Matching variables
Bai et al., 2022 (29)	USA, UK, and Australia	Migraine (physician diagnosis)	P: 35 C: 341	11.5 ± 3.8	62.9% normal BMI; 21.0% underweight; 16.1% overweight/obese	33.3%		NA
Berlinberg et al., 2021 (30)	USA	Axial spondyloarthritis who met the 2009 Assessment of SpondyloArthritis International Society criteria	P: 21 C: 24	P: 44.92 ± 12.1 C: 45.16 ± 11.8		P: 43.5% C: 50.0%	No antibiotics last 2 weeks, no aspirin or NSAIDs last 7 days, no anticoagulation.	NA
Braundmeier-Fleming et al., 2016 (31)	USA	Interstitial cystitis/bladder pain syndrome	P: 18 C: 16	P: 35 ± 9 C: 35 ± 11		P: 100% C: 100%	No antibiotics in previous 3 months	NA
Chen et al., 2019 (32)	China	Migraine	P: 54 C: 54	P: 61.0 ± 8.4 C: 62.5 ± 9.6	P: 26.2 ± 4.6 C: 25.4 ± 3.35	P: 100% C: 100%		Age and BMI
Clos-Garcia et al., 2019 (33)	Spain	FM who met the 2016 diagnostic criteria	P: 105 C: 54	P: 52.52 ± 10.3 C: 53.5 ± 12.4		P: 69.52% C: 48.15%	P: 70% painkillers; 55% antidepressants/benzodiazepines; 30% antiepiliptic drugs	Age and same environment
Frémont et al., 2013 (34)	Belgium and Norway	ME/CFS who met Fukuda criteria	P Belgium: 18 P Norway: 25 C Belgium: 19 C Norway: 17	P Belgium: 38.5 (13) P Norway: 41 (12.5) C Belgium: 41 (12.6) C Norway: 45 (19)		P Belgium: 83.3% P Norway: 88% C Belgium: 78.95% C Norway: 82.3%	No use of antibiotics or probiotics for four weeks prior to sample collection.	NA
Giloteaux et al., 2016 (35)	USA	ME/CFS who met Fukuda criteria	P: 49 C: 39	P: 50.2 (12.6) C: 45.5 (9.9)	P: 25.5 (4.9) C: 27.1 (6.1)	P: 77.5% C: 76.9%		NA
Guo et al., 2023 (36)	USA	ME/CFS cases who met 1994 CDC and 2003 Canadian consensus criteria	P: 106 C: 91	P: 47.8 ± 13.7 C: 47.0 ± 14.1	P: 26.1 ± 5.2 C: 25.2 ± 4.7	P: 70.8% C: 75.8%	P: 22.6% painkillers, 12.3% antibiotics and 38.7% antidepressants C: 2.2% painkillers, 5.5% antibiotics and 13.2% antidepressants	geographical/clinical site, sex, age, race/ethnicity, and date of sampling ( ± 30 days)
Janulewicz et al., 2019 (37)	USA	Gulf War Illness fulfilling Kansas GWI case criteria	P: 3 C: 5	P: 63.2 ± 15.5 C: 52.8 ± 6.7	P: 31.9 ± 0.7 C: 28.6 ± 2.5	P: 33.3% C: 0%		NA
Kitami et al., 2020 (38)	Japan	ME/CFS who met Fukuda criteria in 1994, International Consensus Criteria, and Systemic Exertion Intolerance Disease criteria	P: 48 (28 microbiome data) C: 52 (39 microbiome data)	P: 37 (33-42) C: 40 (34-45)	P: 21 (19-23) C: 20 (19.8-22)	P: 85.4% C: 90.4%		Age, gender, and BMI
Kopchak et al., 2022 (39)	Ukraine	Chronic and Episodic forms of migraine	P+C: 100	P+C: 38.6 ± 8		P+C: 85.3%		NA
Lupo et al., 2021 (40)	Italy	ME/CFS who met Fukuda criteria	P: 35 C: 35	P: 46.4 (16.1) C: 55.2 (18)	P: 23.1 (4.4) C: 23.5 (4.7)	P: 74.3% C: 74.3%	No use of antibiotics, cortisone and non-steroidal anti-inflammatory drugs, inhibitors of proton pump inhibitors and probiotic drugs in the two months before the study.	Age, sex and BMI
Mandarano et al., 2018 (41)	USA	ME/CFS who met Fukuda criteria in 1994	P: 17 (11 for alpha and beta diversity) C: 17 (10 for alpha and beta diversity)	P: 52 (11.9) C: 44.6 (10.9)	P: 26.8 (4.7) C: 27.4 (4.5)	P: 76.47% C: 94.12%		NA
Minerbi et al., 2019 (42)	Canada	FM who met the 2016 diagnostic criteria	P: 77 C: 79	P: 46 ± 8		P: 100%	No antibiotics in previous 2 months	NA, however, controls include first-degree relatives, household members, and unrelated women.
Nagy-Szakal et al., 2017 (43)	USA	ME/CFS who met the 1994 CDC Fukuda and the 2003 Canadian consensus criteria	P: 50 C: 50	P: 51.081 SEM± 1.607 C: 51.320 SEM± 1.620	P: 56% BMI < 25kg/m² and 44% <25 kg/m² C: 44% BMI < 25kg/m² and 56% <25 kg/m²	P: 82% C: 82%		Age, sex, race/ethnicity, geographic/clinical site and season of sampling
Reichenberger et all., 2013 (44)	USA	CRPS who met IEASP criteria (87.5% Type 1)	P: 11 (no GI symptoms) C: 16	P: 40.45 C: 35.63	P: 25.70 ± 1.65 C: 23.68 ± 0.70	P: 100% C: 100%	No antibiotics or narcotics previous 3 months. P: 63% Antiepileptics; 57% antidepressants; 31% antianxiolytics.	NA
Sheedy et al., 2009 (45)	Australia	CFS who met Holmes, Fukuda and Canadian Definition Criteria	P: 108 C: 177					NA
Shukla et al., 2015 (46)	USA	ME/CFS who met Fukuda criteria in 1994	P: 10 C: 10	P: 48.6 ± 10.5 C: 46.5 ± 13.0	P: 23.9 ± 4.3 C: 24.6 ± 3.3	P: 80% C: 80%	No opioids or immunomodulatory medications, antibiotics, probiotics.	Age, gender, BMI, and self-reported general activity patterns
Weber et al., 2022 (47)	Austria	FM who met the 2016 American College of Rheumatology criteria	P: 25 C: 26	P: 49.8 ± 8.6 C: 50.0 ± 8.0	P: 25.6 ± 5.6 C: 23.8 ± 4.0	P: 88% C: 81%	P: 68% NSAID, 36% antidepressants, 20% antihypertensive drugs; 24% proton pump inhibitors; 12% antibiotics; 40% tetrahydrocannabinol/cannabidiol C: 31% NSAID, 8% antidepressants, 8% antihypertensive drugs; 8% proton pump inhibitors	Age and sex
Yong et al., 2023 (48)	Korea	Episodic migraine (P1) and Chronic migraine (P2) who fulfilled ICHD-3 criteria of EM (code 1.1 or 1.2) or CM (code 1.3)	P1: 42 P2: 45 C: 43	P1: 39.6 ± 11.4 P2: 40.8 ± 12.5 C: 43.2 ± 11.7	P1: 22.8 ± 2.5 P2: 22.7 ± 3.5 C: 22.1 ± 3.6	P1: 78.6% P2: 91.1% C: 81.4%	P1: 47.6% anti-epileptic medication, 26.2% beta blockers, 4.8% anti-depressant, 2.4% calcium-channel blocker. P2: 51.1% anti-epileptic, 17.8% beta blockers, 2.2% anti-depressant.	Age, sex, BMI
Zhao et al., 2021 (49)	China	Chronic stable angina who met American College of Cardiology/American Heart Association criteria	P: 30 C: 10	P: 62 (Q1-Q3: 41-80) C: 60 (Q1-Q3: 40-76)	P: 22.5 (Q1-Q3: 18.4-24.1) C: 22.3 (Q1-Q3: 20.8-23.5)	P: 43.33% C: 50%	P: 100% beta-blockers; 100% long-lasting nitrates; 3.3% ACE inhibitors; 20% calcium channel blockers; 6.7% angiotensin receptor blockers	NA

BMI, body mass index; C: controls; ME/CFS, myalgic encephalomyelitis/chronic fatigue syndrome; NA, not applicable; P, patients.

### 3.3 Microbiome characteristics

After collection of samples, 14 studies (66.7%) froze the samples at -80°C until further use, 1 study (4.8%) at -70°C, 2 studies (9.5%) at -20°C, and it was not reported for 4 studies (19%). In terms of stool processing, a broad variety was observed (**Supplementary Table 2**). Only one study explored eukaryotes (41). In terms of sequencing, 14 studies conducted 16S sequencing, 3 studies shotgun metagenomics, 1 study paired-end metagenomic sequencing, 1 study 18S sequencing, and 2 studies did not report the sequencing. The 18S sequencing was performed at region V9, while the 16S sequencing was performed at regions V1-V2 (1 study), V2 (1 study), V3-V4 (4 studies), V3-V5 (1 study), V4 (4 studies), and V5-V6 (2 studies).

#### 3.3.1 Alpha-diversity

**Figure 2:**
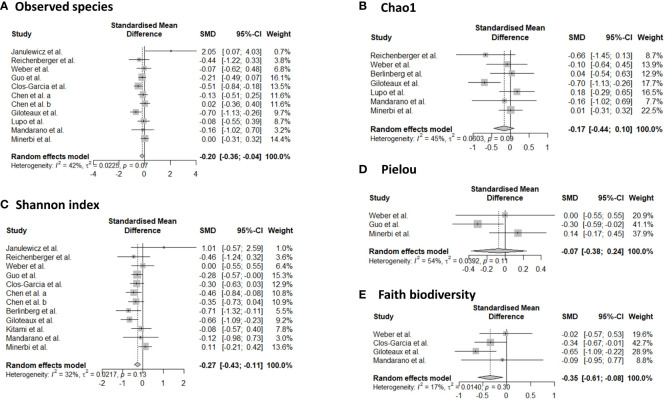
Forest plots of α-diversity metrics observed species **(A)**, Chao1 **(B)**, Shannon index **(C)**, Pielou **(D)**, and faith phylogenetic diversity **(E)**. Standardized mean differences were used as effect sizes whereby a negative point estimate denotes a higher value in controls and a positive estimate a higher value in chronic pain patients.

Sixteen studies provided data for alpha-diversity, evaluated through 8 different metrics. When evaluating richness through observed species, non-significant differences were revealed for patients with axial spondyloarthritis (30), ME/CFS (36, 40, 41), migraine (32), and fibromyalgia (33, 47) compared to controls. For patients with ME/CFS, only one study found significant differences with higher richness in controls compared to patients (35). Significantly increased values for observed species were found for patients with Gulf War Illness (37), while significantly decreased values for patients with CRPS (44) in relation to controls. Based on pooled estimates, a significant SMD of -0.201 (95% CI from -0.04 to -0.36, p=0.01, I²=41.9%, 11 effect sizes) was revealed, classified as a small effect size, pointing towards lower observed species numbers in chronic pain patients compared to controls (**Figure 2**). Egger’s test did not reveal indications for funnel plot asymmetry (t=0.9, df=9, p=0.39). For Chao1, significantly reduced values were obtained in patients with CRPS (44) and in one study with ME/CFS patients (35), while the other studies did not reveal significant differences between chronic pain patients and controls (30, 33, 40, 41, 47, 48). Non-significant results were revealed for the abundance coverage estimator (33, 47), as confirmed with a meta-analysis (SMD of -0.17 (95% CI from -0.44 to 0.10), p=0.22). For evenness, the Pielou metric resulted in significantly lower values in patients with ME/CFS compared to controls (36), while other reports did not reveal significant differences (29, 47). For richness/evenness, 15 studies explored the Shannon index with significant differences in favor of chronic pain patients (37), in favor of controls (29, 32, 35, 36, 44), and no significant difference between controls and chronic pain patients (30, 33, 34, 38, 41, 42, 47, 48). A random-effect meta-analysis resulted in a significantly decreased index in chronic pain patients compared to controls (p<0.001) with a small effect size (SMD -0.27, 95% CI from -0.11 to -0.43, 12 effect sizes, Egger’s Test t=0.25, df=10, p=0.81). Non-significant results were revealed for the Simpson index between chronic pain patients and controls (30, 33, 38, 40, 48), as was the case for the inverse Simpson index (42, 47). Faith phylogenetic diversity indicated increased values in controls in three studies (29, 33, 35), while two other studies revealed no significant differences (41, 47) between chronic pain patients and controls. A random-effect meta-analysis resulted in a significantly decreased index in chronic pain patients compared to controls (p=0.01) with a small effect size (SMD -0.35, 95% CI from -0.08 to -0.61, 4 effect sizes, Egger’s Test t=0.71, df=2, p=0.55). The meta-analysis for Chao1 and Pielou did not reveal significant differences between controls and chronic pain patients. The study of Zhao et al. (49) provided mean values for observed species, Chao1, abundance coverage, Shannon index, and Simpson index for patients with chronic stable angina compared to controls, however, it was not clear whether the results were significant. Therefore, these results were not qualitatively discussed, however, they are incorporated into the meta-analyses.

#### 3.3.2 Beta-diversity

Ten studies explored beta-diversity with the aid of three different metrics (Bray-Curtis, Weighted UniFrac, and Unweighted UniFrac) (29, 30, 35, 36, 41–44, 48, 49). In patients with migraine, inconsistent results were revealed with significant differences in beta-diversity according to Bai et al. (Bray-Curtis and Weighted UniFrac) (29) and non-significant results by Yong et al. (Bray-Curtis, Weighted UniFrac, and Unweighted UniFrac) (48). In patients with ME/CFS, two studies pointed towards significant differences in β-diversity, measured with Bray-Curtis, compared to healthy participants (36, 43), and two other studies did not reveal differences (35, 41). For patients with fibromyalgia (42), CRPS (44), and chronic stable angina (49), significant differences in beta-diversity were revealed, by one study for each condition. A non-significant result was revealed for patients with axial spondyloarthritis (30).

#### 3.3.3 Differentially abundant microbes

Twenty out of twenty-one studies explored the relative abundance of gut microbes in chronic pain patients compared to controls (**Table 3**). Differences were found in 8 phyla, 14 families, 52 genera, and 73 species. An overview of the differences between the populations can be found in **Table 4**. At the phylum level, four main taxa were explored namely Actinobacteria (29, 33, 46), Bacteroidetes (29, 33, 40, 46), Firmicutes (29, 32, 33, 35, 40, 44, 46, 49), and Proteobacteria (29, 35, 44, 49). For Actinobacteria, Bacteroidetes, and Firmicutes both increases and decreases were revealed in chronic pain patients compared to controls, pointing towards inconsistent results. For Proteobacteria, a decrease was revealed in chronic pain patients compared to controls in all four studies (29, 35, 44, 49). Four fungal phyla were explored as well, with an increase in abundance in controls in Ascomycotae and decreased abundances in Basidiomycotae, Stramenopiles, and Zygomycota (41). At the family level, Lachnospiraceae were most often explored whereby 5 out of 6 studies indicated a decrease in relative abundance in chronic pain patients, compared to controls (29, 33, 37, 40, 43). At the genus level, *Faecalibacterium* spp. were most often explored, followed by *Dorea* spp., *Eggerthella* spp., and *Roseburia* spp. A decrease was found in *Faecalibacterium* spp. in patients with migraine (32, 48), ME/CFS (35, 38, 43) and chronic angina (49). For *Dorea* spp., inconsistent results were revealed for migraine patients (29, 48), an increase in patients with FM (33), and a decrease in patients with ME/CFS compared to controls (43). For *Roseburia* spp., 3 out of 4 studies revealed an increased relative abundance in controls (34, 43, 48), while one study revealed an increase in patients with fibromyalgia (33). In the genus *Eggerthella*, an increased relative abundance was found in patients with migraine (29, 48) and ME/CFS (35, 38). At the species level, a decrease in the relative abundance of *Faecalibacterium prausnitzii* was revealed for patients with migraine (32), ME/CFS (36, 43), fibromyalgia (42), and bladder pain syndrome (31). *Odoribacter splanchnicus* had a lower abundance in patients with migraine (32), ME/CFS (43), and bladder pain syndrome (31). *Clostridium asparagiforme* and *Clostridium symbiosum* increased in patients with migraine and ME/CFS, while *Coprococcus catus* and *Ruminococcus obeum* decreased in these patients (32, 43). *Flavonifractor plautii* had an increased abundance in patients with migraine and fibromyalgia (32, 42). Finally, *Eggerthella lenta* also increased in patients with migraine (32, 39).

**Table 3:** Composition analysis of the included studies.

Author	OTU	Chao1	Abundance coverage	Evenness	Shannon	Simpson	Inverse Simpson	Faith	Beta Diversity	Relative abundance
Bai et al., 2022 (29)				NS	C higher than P			C higher than P	Bray-Curtis: S Weighted UniFrac: S	Phylum level: Higher Bacteroidetes, Actinobacteria, Firmicutes, Probacteria in P. Firmicutes also higher in C. Family level: Higher unidentified family Lachnospiraceae, unidentified family Erysipelotrichaceae in P than C. Higher unidentified family Christensenellaceae, unidentified family Lachnospiraceae, and unidentified family Ruminococcaceae in C. Genus level: Higher *Bacteroides, Parabacteroides*, and *Odoribacter, Eggerthella* and *Varibaculum, SMB53, Lachnospira, Dorea, Veillonella, Anaerotruncus, Butyricicoccus, Eubacterium, Coprobacillus, Sutterella* in P than C. Higher *Anaerostipes* and *Oribacterium* in C.
Berlinberg et al., 2021 (30)	NS	NS			NS	NS			Bray-Curtis: NS	Species level: Higher *Bifidobacterium adolescentis* and *Porphyromonas bennonis* in P. Higher *Streptococcus anginosus* and *Bacteroides dorei* in C.
Braundmeier-Fleming et al., 2016 (31)										Species level: lower *E. sinensis*, *C. aerofaciens, F. prausnitzii, O. splanchnicus*, and *L. longoviformis* in P.
Chen et al., 2019 (32)	NS at genus and species level				Decreased in P compared to C					Phylum level: higher Firmicutes in P. Genus level: lower Faecalibacterium in P. Species level: higher *Faecalibacterium* *prausnitzii, Bifidobacteriumadolescentis*, and *Methanobrevibacter smithii* in C. Higher *Blautia hydrogenotrophica, Clostridium asparagiforme, Clostridium clostridioforme, Clostridium bolteae, Clostridium citroniae, Clostridium hathewayi, Clostridium ramosum*, *Clostridium spiroforme, Clostridium symbiosum, Eggerthella lenta, Flavonifractor plautii, Lachnospiraceae bacterium*, and *Ruminococcus gnavus* in P. Higher *Bacteroides clarus, Bacteroides intestinalis, Bacteroides salyersiae, Bacteroides stercoris, Butyrivibrio crossotus, Clostridium* sp. *L2_50, Coprococcus catus, Eubacterium hallii, Eubacterium ramulus, Odoribacter splanchnicus, Peptostreptococcaceae* *noname unclassified, Prevotella copri, Ruminococcus callidus, Ruminococcus champanellensis, Ruminococcus obeum*, and *Sutterella wadsworthensis* in C.
Clos-Garcia et al., 2019 (33)	NS	NS	NS		NS	NS		P lower than C		Phylum level: Bacteroidetes and Firmicutes both increased and decreased, Actinobacteria reduced in P. Family level: Higher Rikenellaceae in P. Lower unassigned genus in Bacteroidaceae and Lachnospiraceae families, Bifidobacteriaceae and Erysipelotichaceae in P. Genus level: Lower *Bacteroides, Bifidobacterium, Eubacterium* and *Clostridium* in P. Higher *Dorea, Roseburia* and *Alistipes* in P.
Frémont et al., 2013 (34)					NS					Genus level: Higher *Lactonifactor* and *Alistipes* in Norwegian P. Higher *Roseburia, Syntrophococcus, Holdemania* and *Dialister* in Norwegian C. Higher *Lactonifactor* in Belgian P. Higher *Asaccharobacter* in Belgian C.
Giloteaux et al., 2016 (35)	C: 1486.5 (456.5) P: 1204.3 (351.2) S	C: 2918.4 (884.9) P: 2363.5 (705) S			C: 5.9 (0.9) P: 5.3 (0.9) S			C: 73.4 (19.0) P: 61.7 (16.7) S	Weighted UniFrac: NS Unweighted UniFrac: NS	Phylum level: lower Firmicutes in P. Higher Proteobacteria in P. Family level: higher Enterobacteriaceae, Prevotellaceae in P. Lower Ruminococcaceae, Bacteroidaceae, Rickenellaceae, Bifidobacteriaceae in P. Genus level: Higher *Oscillospira, Lactococcus, Anaerotruncus, Coprobacillus* and *Eggerthella* in P. Higher *Faecalibacterium* and *Bifidobacterium* in C.
Guo et al., 2023 (36)	NS			P lower then C	P lower then C				Bray-Curtis: S	Species level: Lower *F. prausnitzii, E. rectale*, and *C. secundus* in P. Higher *R. lactatiformans, C. bolteae, R. gnavus, E. ramosum, C. scindens, Blauti* sp. *N6H1.15, S. intestinalis, T. nexilis*, and *Lachnoclostridium* sp. *YL32* in P.
Janulewicz et al., 2019 (37)	P: 576 (SD: 12.9) C: 415 (SD: 83.1) S				P: 4.03 (SD: 0.15) C: 3.79 (SD: 0.23) S (family level)					Phylum level: NS Family level: higher Lachnispiracae in C compared to P. Genus level: Higher *Dialister* in C than P. *Ruminococcus* higher in P than C.
Kitami et al., 2020 (38)					NS	NS				Genus level: Higher *Blautia, Coprobacillus, Eggerthella* in P. Higher *Collinsella, Faecalibacterium* and *Lachnospira* in C.
Kopchak et al., 2022 (39)										Species level: higher frequency of *Alceligenes spp, Clostridium coccoides, Clostridium propionicum, Eggerthella lenta, Pseudonocardia spp, Rhodococcus spp, Micromycetes spp (campesterol* and *sitosterol), Herpes simplex* for P than C.
Lupo et al., 2021 (40)	P: 215.6 (78) C: 221.4 (60.8) NS	P: 453.4 (194.7) C: 422 (151.2) NS				P: 17.7 (11.1) C: 13.3 (7.3) NS				Phylum level: Higher Bacteroidetes in P. Higher Firmicutes in C. Class level: Higher Bacteroidia in P. Higher Clostridia in C. Order level: Higher Clostridiales in C. Higher Bacteroidales in P. Family level: Lower Lachnospiraceae in P. Higher Bacteroidaceae, Barnesiellaceae in P. Genus level: Lower *Anaerostipes* in P. Higher *Bacteroides* and *Phascolarctobacterium* in P. Species level: Higher *Bacteroides ovatus* and *Bacteroides uniformis* in P.
Mandarano et al., 2018 (41)	C: 18.1 (SE: 6.9) P: 14.1 (SE: 7.8) NS	C: 26.6 (SE: 10.7) P: 20.6 (SE: 10.9) NS			C: 2.8 (SE: 1.2) P: 2.3 (SE: 1.2) NS			C: 6.7 (SE:2.1) P: 6.0 (SE:2.4) NS	Weighted UniFrac: NS Unweighted UniFrac: NS	Phylum level fungi: lower Ascomycota in P. Higher Basidiomycota, Stramenopiles and Zygomycota in P. Class level: higher Agaricomycetes, Tremellomycetes in P. Order level: lower Saccharomycetales in P. Higher Agaricales, Boletales, Polyporales, Tremellomycetes unknown, Malasseziales, Entomophthorales, Mucorales, Pleurosigma, Eustigmatales, Peronosporales, Cystofilobasidiales in P. Tremellales, Sporidiobolales and Ustilaginales only observed in C. Species level: Higher *Blastocystis* in P.
Minerbi et al., 2019 (42)					NS		NS		Bray-Curtis: S	Species level: Lower *F. prausnitzii* and *B. uniformis* in P. Higher *Intestinimonas butyricipro ducens, Flavonifractor plautii, Butyricoccus desmolans, Eisenber giella tayi*, and *Eisenbergiella massiliensis* in P.
Nagy-Szakal et al., 2017 (43)									Bray-Curtis: C lower than P.	Family level: lower Lachnospiraceae and Porphyromonadaceae in P, while higher Clostridiaceae. Genus level: lower *Dorea, Faecalibacterium*, *Coprococcus, Roseburia*, and *Odoribacter* in P, while higher *Clostridium* and *Coprobacillus*. Species level: lower *Faecalibacterium prausnitzii, Faecalibacterium cf., Roseburia inulinivorans, Dorea longicatena, Dorea formicigenerans, Coprococcus catus, Odoribacter splanchnicus, Ruminococcus obeum*, and *Parabacteroides merdae* in P, while higher *Clostridium asparagiforme, Clostridium symbiosum*, and *Coprobacillus* bacterium in P.
Reichenberger et all., 2013 (44)	P: mean 280.45 (195-392 range) C: mean 328.63 (145-591 range) S	P: 520.76 (SE: 44.18) C: 651.75 (SE: 54.12) S			P: 3.89 (SE: 0.15) C: 4.12 (SE: 0.12) S				Unweighted UniFrac matrix: S (since clustering is successful based on disease state)	Phylum level: Firmicutes 64.8% in C and 44% in P, Proteobacteria 0.078% in C and 5.1% in P.
Sheedy et al., 2009 (45)										Species level: Higher *E. Coli* in C. Higher *E. faecalis, S. sanguinis* in P.
Shukla et al., 2015 (46)										Phylum level: Higher Bacteroidetes (P 27.71% vs C 22.43%), lower Firmicutes (P 58.40% vs 65.29%) and lower Actinobacteria (P 0.58% vs C 1.06%) in P.
Weber et al., 2022 (47)	P: 194.85 (SD: 42.98) C: 197.99 (SD: 49.69) NS	P: 183.37 (50.84) C: 187.96 (44.04) NS	P: 212.46 (139.9) C: 185.53 (41.58) NS	P: 0.73 (0.05) C: 0.73 (0.05) NS	P: 5.58 (0.56) C: 5.58 (0.56) NS		P: 0.15 (0.04) C: 0.14 (0.05) NS	P: 16.07 (SD:2.71) C: 16.13 (2.98) NS		
Yong et al., 2023 (48)		NS			NS	NS			Weighted UniFrac: NS Unweighted UniFrac: NS Bray-Crutis: NS	Phylum level: no difference. Class level: Higher Tissierellia in P1 and P2 than C. Order level: Higher Tissierellales in P1 and P2 than C. Family level: Higher Peptoniphilaceae and Eubacteriaceae in P1 than C. Higher Peptoniphilaceae in P2 than C. Genus level: Higher *Olsenella* in P1 than C. Higher *Hungatella*, *Clostridium_g6, Eggerthella* and *Longicatena* in P2 than C. Higher *Catenibacterium, PAC000195_g, Fusicantenibacter, Agathobacter, Eubacterium_g4, Roseburia, Lachnospiraceae_uc, Eubacterium_g21* in C than P1. Higher *PAC001134_g, Catenibacterium, PAC000692_g, Holdemanella, PAC001137_g, PAC000195_g, Agathobacter, Eubacterium_g4, Roseburia, Frisingicoccus, Faecalibacterium, Dorea* and *Lachnospira* in C than P2.
Zhao et al. (49)	P: 323.05 C: 321.9	P: 327.86 C: 327.51	P: 336.72 C: 335.62		P: 5.26 C: 5.84	P: 0.91 C: 0.96			Weighted UniFrac: S	Phylum level: lower Firmicutes in P, and higher Probacteria in P. Genus level: higher *Anaerostipes, Erysipelatoclostridium, Holdemanella, Sarcina, Streptococcus*, and *Weissella* in P. Lower *Faecalibacterium, Romboutsia*, and *Subdoligranulum* in P.

C, controls; NA, not applicable; NS, non-significant; P, patients; S, significant.

**Table 4:** Changes in relative abundance of microbes in chronic pain patients compared to controls at phylum, family, genus and species level.

	Migraine	ME/CFS	FM	Axial spondy-oarthritis	Bladder pain syndrome	Gulf-war	CRPS	Chronic angina
Phylum level								
Actinobacteria	Higher P (29)	Higher C (46)	Higher C (33)					
Bacteroidetes	Higher P (29)	Higher P (46) Higher P (40)	Higher P (33) Higher C (33)					
Firmicutes	Higher C (29) Higher P (29) Higher P (32)	Higher C (46) Higher C (35) Higher C (40)	Higher P (33) Higher C (33)				Higher C (44)	Higher C (49)
Proteobacteria	Higher P (29)	Higher C (35)					Higher P (44)	Higher P (49)
Ascomycota		Higher C (41)						
Basidiomycota		Higher P (41)						
Stramenopiles		Higher P (41)						
Zygomycota		Higher P (41)						
Family level								
Bacteroidaceae		Higher C (35) Higher P (40)	Higher C (33)					
Barnesiellaceae		Higher P (40)						
Bifidobacteriaceae		Higher C (35)	Higher C (33)					
Christensenellaceae	Higher C (29)							
Clostridiaceae		Higher P (43)						
Erysipelotrichaceae	Higher P (29)		Higher C (33)					
Enterobacteriaceae		Higher P (35)						
Eubacteriaceae	Higher P (48)							
Lachnospiraceae	Higher P (29) Higher C (29)	Higher C (43) Higher C (40)	Higher C (33)			Higher C (37)		
Peptoniphilaceae	Higher P (48)							
Porphyromonadaceae		Higher C (43)						
Prevotellaceae		Higher P (35)						
Rikenellaceae		Higher C (35)	Higher P (33)					
Ruminococcaceae	Higher C (29)	Higher C (35)						
Genus level								
*Agathobacter*	Higher C (48)							
*Alistipes*		Higher P (34)	Higher P (33)					
*Anaerostipes*	Higher C (29)	Higher C (40)						Higher P (49)
*Anaerotruncus*	Higher P (29)	Higher P (35)						
*Asaccharobacter*		Higher C (34)						
*Bacteroides*	Higher P (29)	Higher P (40)	Higher C (33)					
*Bifidobacterium*		Higher C (35)	Higher C (33)					
*Blautia*		Higher P (38)						
*Butyricicoccus*	Higher P (29)							
*Catenibacterium*	Higher C (48)							
*Clostridium*	Higher P (48)	Higher P (43)	Higher C (33)					
*Collinsella*		Higher C (38)						
*Coprobacillus*	Higher P (29)	Higher P (43) Higher P (38)						
*Coprococcus*		Higher C (43) Higher P (35)						
*Dialister*		Higher C (34)				Higher C (37)		
*Dorea*	Higher C (48) Higher P (29)	Higher C (43)	Higher P (33)					
*Eggerthella*	Higher P (29) Higher P (48)	Higher P (38) Higher P (35)						
*Erysipelatoclostridium*								Higher P (49)
*Eubacterium*	Higher C (48) Higher P (29)		Higher C (33)					
*Faecalibacterium*	Higher C (48) Higher C (32)	Higher C (43) Higher C (38) Higher C (35)						Higher C (49)
*Frisingicoccus*	Higher C (48)							
*Fusicantenibacter*	Higher C (48)							
*Holdemanella*	Higher C (48)							Higher P (49)
*Holdemania*		Higher C (34)						
*Hungatella*	Higher P (48)							
*Lachnospira*	Higher P (29) Higher C (48)	Higher C (38)						
*Lachnospiraceae_uc*	Higher C (48)							
*Lactococcus*		Higher P (35)						
*Lactonifactor*		Higher P (34)						
*Longicatena*	Higher P (48)							
*Odoribacter*	Higher P (29)	Higher C (43)						
*Olsenella*	Higher P (48)							
*Oribacterium*	Higher C (29)							
*Oscillospira*		Higher P (35)						
*PAC000195_g*	Higher C (48)							
*PAC000692_g*	Higher C (48)							
*PAC001134_g*	Higher C (48)							
*PAC001137_g*	Higher C (48)							
*Parabacteroides*	Higher P (29)							
*Phascolarctobacterium*		Higher P (40)						
*Romboutsia*								Higher C (49)
*Roseburia*	Higher C (48)	Higher C (43) Higher C (34)	Higher P (33)					
*Ruminococcus*						Higher P (37)		
*Sarcina*								Higher P (49)
*SMB53*	Higher P (29)							
*Streptococcus*								Higher P (49)
*Subdoligranulum*								Higher C (49)
*Sutterella*	Higher P (29)							
*Syntrophococcus*		Higher C (34)						
*Varibaculum*	Higher P (29)							
*Veillonella*	Higher P (29)							
*Weissella*								Higher P (49)
Species level								
*Alceligenes spp*	Higher P (39)							
*B. Uniformis*			Higher C (42)					
*Bacteroides clarus*	Higher C (32)							
*Bacteroides dorei*				Higher C (30)				
*Bacteroides intestinalis*	Higher C (32)							
*Bacteroides ovatus*		Higher P (40)						
*Bacteroides salyersiae*	Higher C (32)							
*Bacteroides stercoris*	Higher C (32)							
*Bacteroides uniformis*		Higher P (40)						
*Bifidobacterium adolescentis*	Higher C (32)			Higher P (30)				
*Blastocystis*		Higher P (41)						
*Blauti* sp. *N6H1.15*		Higher P (36)						
*Blautia hydrogenotrophica*	Higher P (32)							
*Butyricoccus desmolans*			Higher P (42)					
*Butyrivibrio crossotus*	Higher C (32)							
*C. aerofaciens*					Higher C (31)			
*C. bolteae*		Higher P (36)						
*C. scindens*		Higher P (36)						
*C. secundus*		Higher C (36)						
*Clostridium asparagiforme*	Higher P (32)	Higher P (43)						
*Clostridium bolteae*	Higher P (32)							
*Clostridium citroniae*	Higher P (32)							
*Clostridium clostridioforme*	Higher P (32)							
*Clostridium coccoides*	Higher P (39)							
*Clostridium hathewayi*	Higher P (32)							
*Clostridium propionicum*	Higher P (39)							
*Clostridium ramosum*	Higher P (32)							
*Clostridium* sp. *L2_50*	Higher C (32)							
*Clostridium spiroforme*	Higher P (32)							
*Clostridium symbiosum*	Higher P (32)	Higher P (43)						
*Coprobacillus bacterium*		Higher P (43)						
*Coprococcus catus*	Higher C (32)	Higher C (43)						
*Dorea formicigenerans*		Higher C (43)						
*Dorea longicatena*		Higher C (43)						
*E. coli*		Higher C (45)						
*E. faecalis*		Higher P (45)						
*E. ramosum*		Higher P (36)						
*E. rectale*		Higher C (36)						
*E. sinensis*					Higher C (31)			
*Eggerthella lenta*	Higher P (32) Higher P (39)							
*Eisenber giella tayi*			Higher P (42)					
*Eisenbergiella massiliensis*			Higher P (42)					
*Eubacterium hallii*	Higher C (32)							
*Eubacterium ramulus*	Higher C (32)							
*Faecalibacterium cf.*		Higher C (43)						
*Faecalibacterium prausnitzii*	Higher C (32)	Higher C (36) Higher C (43)	Higher C (42)		Higher C (31)			
*Flavonifractor plautii*	Higher P (32)		Higher P (42)					
*Herpes simplex*	Higher P (39)							
*Intestinimonas butyricipro ducens*			Higher P (42)					
*L. longoviformis*					Higher C (31)			
*Lachnoclostridium* sp. *YL32*		Higher P (36)						
*Lachnospiraceae bacterium*	Higher P (32)							
*Methanobrevibacter smithii*	Higher C (32)							
*Micromycetes spp (campesterol and sitosterol)*	Higher P (39)							
*Odoribacter splanchnicus*	Higher C (32)	Higher C (43)			Higher C (31)			
*Parabacteroides merdae*		Higher C (43)						
*Peptostreptococcaceae*	Higher C (32)							
*Porphyromonas bennonis*				Higher P (30)				
*Prevotella copri*	Higher C (32)							
*Pseudonocardia spp*	Higher P (39)							
*R. gnavus*		Higher P (36)						
*R. lactatiformans*		Higher P (36)						
*Rhodococcus spp*	Higher P (39)							
*Roseburia inulinivorans*		Higher C (43)						
*Ruminococcus callidus*	Higher C (32)							
*Ruminococcus champanellensis*	Higher C (32)							
*Ruminococcus gnavus*	Higher P (32)							
*Ruminococcus obeum*	Higher C (32)	Higher C (43)						
*S. intestinalis*		Higher P (36)						
*S. sanguinis*		Higher P (45)						
*Streptococcus anginosus*				Higher C (30)				
*Sutterella wadsworthensis*	Higher C (32)							
*T. nexilis*		Higher P (36)						

C, controls; P, patients.

## 4 Discussion

This study evaluated alterations in gut microbiota composition in chronic pain patients compared to controls. In terms of alpha-diversity, the richness metric observed species indicated a significantly decreased number of unique operational taxonomic units in chronic pain patients. Additionally, a lower Shannon index and faith phylogenetic diversity were revealed in patients compared to controls. For beta-diversity, inconclusive results were revealed. Finally, there was a decreased relative abundance of Lachnospiraceae in 83% of studies that evaluated this family in chronic pain patients compared to controls. A decreased abundance of *Faecalibacterium prausnitzii* and *Odoribacter splanchnicus* species was demonstrated in patients compared to controls. Based on this systematic review, with complementary meta-analyses, there are indications for dysbiosis of gut microbiota in chronic pain patients.

The interest in gut microbiota as a potential underlying factor of disease maintenance has drastically increased during the last decade. Gut dysbiosis is expected to contribute to the etiology of, e.g., inflammatory bowel disease (50, 51), type 2 diabetes (52), colorectal cancer (53, 54), hypertension (55), and rheumatic diseases (23), besides its modulating role in chronic pain (56). The mechanisms by which acute infectious pain becomes chronic are very diverse and can include, among others, molecular mimicry (structural similarity between microbial and host molecules which could induce autoimmune responses), bystander activation, or microbe invasion (57, 58). Specific microbes such as *Borrelia* species and *Mycobacterium leprae* or viruses (e.g., HIV, SARS-Cov-2) are associated with a high incidence of chronic pain (57). A cross-disease meta-analysis was previously performed, whereby consistent patterns characterizing disease-associated microbiome changes were revealed (59). Some diseases were characterized by the presence of potentially pathogenic microbes, whereas others revealed a depletion of health-associated bacteria (59). About half of the genera associated with individual studies were bacteria that respond to more than one disease, supporting the hypothesis of non-disease-specific alterations but shared alterations (i.e. non-specific response) to health and disease (59). Based on this hypothesis, the current systematic review and meta-analysis was conducted in patients with chronic pain, regardless of the underlying disease condition.

Gut microbiome alpha-diversity has been associated with human health, whereby reduced levels are indicative of acute and chronic diseases (60). Alpha-diversity metrics provide summary statistics that focus on summarizing the breadth of diversity present in an environment (61). The current study indicated a decrease in alpha-diversity in patients with chronic pain compared to controls, as reflected in several metrics namely, a decreased number of unique operational taxonomic units, a decreased Shannon index [which is a popular diversity index in the ecological field to reflect the richness of bacterial community (62)], and a decreased Faith’s phylogenetic diversity in chronic pain patients. Faith’s phylogenetic diversity accounts for the phylogenetic relatedness of community members and has been denoted as more sensitive to distinguishing disease factors relative to other alpha diversity metrics (63). Despite the small effect sizes, these alpha-diversity metrics all point towards a decreased richness in chronic pain patients, which may point out the need for nutritional interventions in patients with chronic pain. The gut microbiota produces polyamines, which in turn excites N-methyl-D-aspartate receptors, a crucial factor of central nervous system sensitization (64), which is common in patients with chronic pain (65).

A reduction in the relative abundance of the Lachnospiraceae family was found in patients with chronic pain. All Lachnospiraceae members are anaerobic, fermentative and chemoorganotrophic, and are already present in early infancy (66). Aging is associated with increases in Lachnospiraceae abundance (67). The genera *Blautia* and *Roseburia*, belonging to the Lachnospiraceae family, are often associated with a healthy state (68). These genera are the main short-chain fatty acid (SCFA) producers [whereby SCFA activity modulates the surrounding microbial environment and interacts with the host immune system (69)] and are involved in the control of gut inflammatory processes, and maturation of the immune system (66, 70). A higher relative abundance of *Roseburia* ssp. was revealed in controls compared to chronic pain patients, highlighting the value of this genus in health states. Additionally, a decrease in the relative abundance of *Odoribacter splanchnicus*, another common SCFA-producing member of the human intestinal microbiota (71), was found in chronic pain patients. This finding was previously also described in patients with inflammatory bowel disease (72, 73).

Another finding was a decreased relative abundance of the Faecalibacterium genus, belonging to the family Ruminococcaceae, which comprises only one validated species, namely *Faecalibacterium prausnitzii* (74). A decrease in *Faecalibacterium prausnitzii* was observed in chronic pain patients, a species known to play a crucial role in host wellbeing and gut physiology (75). It is one of the main butyrate producers in the intestine (76), whereby butyrate is involved in maintaining mucosal integrity, alleviating inflammation (via macrophage function as well as a reduction in proinflammatory cytokines), and increasing anti-inflammatory mediators (77). Thus, this species is known for its anti-inflammatory properties (75). In murine models, it was revealed that *Faecalibacterium prausnitzii* cells could reduce the severity of both acute, chronic, and chemical-induced inflammation (78–80). *Faecalibacterium prausnitzii* depletion has been reported in adults with Crohn’s disease, ulcerative colitis, and colorectal cancer (81–84), as well as in patients with rheumatic disorders (23, 85) and is proposed as a biomarker to discriminate between gut disorders and healthy subjects (75). This alteration may not be specific to inflammatory diseases and may be a more generic phenomenon of disease states since it is also revealed in chronic pain patients.

**Figure 3:**
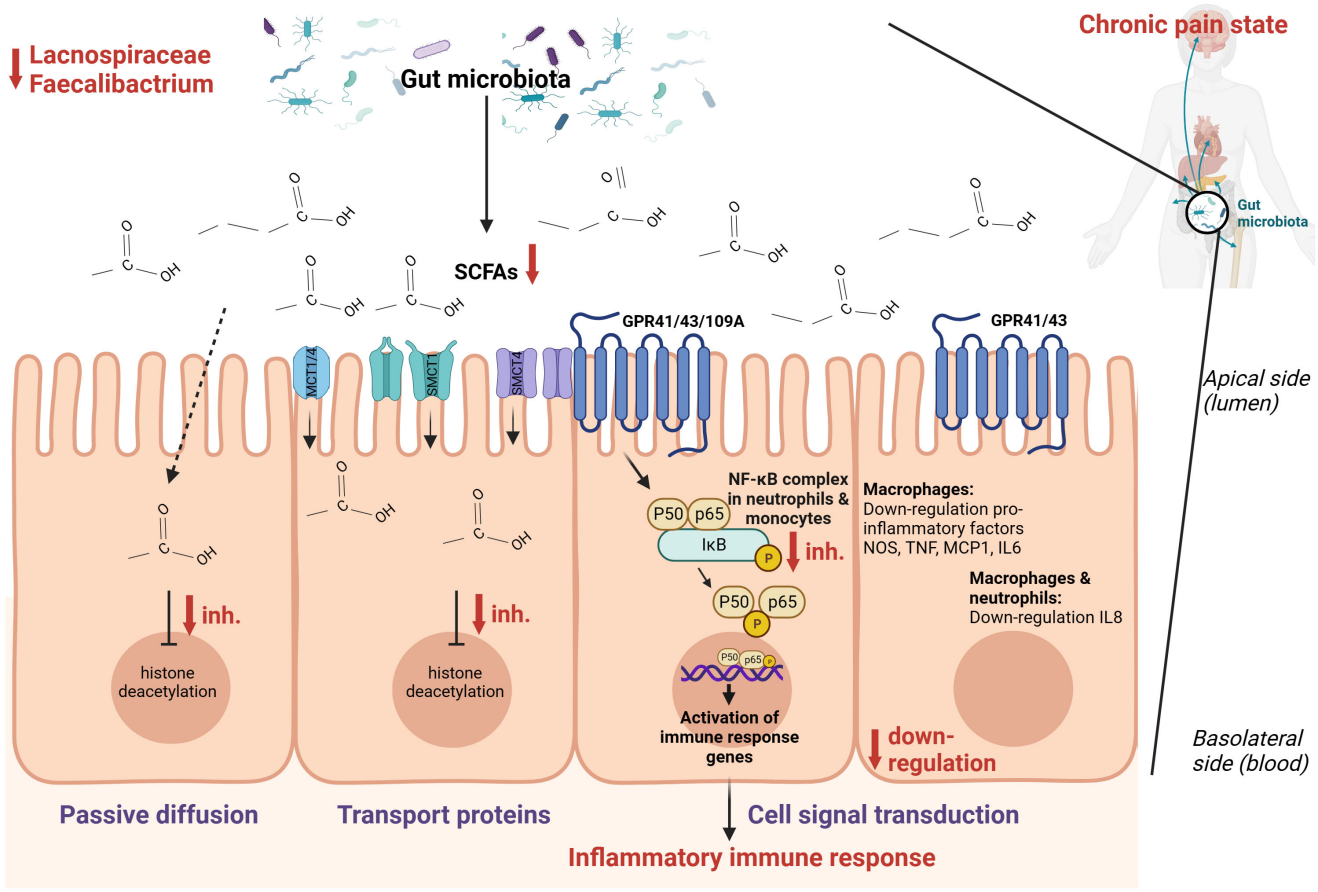
Hypothesized schematic representation of the role of short-chain fatty acids (SCFAs) in the regulation of gut and systemic immunity in relation to chronic pain (86–88). SCFAs can regulate inflammation through cell signal transduction by binding at G-protein coupled receptors GPR109A, GPR43, and GPR41 and down-regulate the NOS, TNF, MCP-1, IL-6, IL-8, and the NF-κB signaling pathway. Through passive diffusion and transport proteins (MCT1, MCT4, SMCT1, SMCT2), SCFAs can inhibit histone deacetylase. This is a simplified representation of the pathways involved in inflammation with the pathways expected to be relevant in the setting of chronic pain.

Combining these findings, it seems that SCFAs [mainly composed of acetic acid, propionic acid, and butyric acid (86)] play an important role in the context of chronic pain (**Figure 3**). There are two main mechanisms through which SCFAs can enter cells and consequently alter inflammation, namely cell signal transduction and passive diffusion combined with transport proteins. The latter functions through sodium-coupled monocarboxylate transport 1/2 (SMCT1/2), Na^+^ coupled transporters in the apical membrane of colonic epithelium, and monocarboxylate transporter 1/4 (MCT1/4), H^+^ coupled transporters mainly expressed in the apical and basolateral membrane of the colonic epithelium (89). Once SCFAs enter the cell through passive diffusion or transporters, they inhibit histone deacetylation (86). In dendritic cells and macrophages, inhibition of histone deacetylation is the main pathway to exert anti-inflammatory effects, while in neutrophils and monocytes, SCFAs inhibit tumor necrosis factor expression, the NF-κB signaling pathway, and histone deacetylase in addition to promoting interleukin-10 production as an anti-inflammatory cytokine. Cell signal transduction is realized by SCFAs through G protein-coupled cell membrane receptors GPR109A, GPR43, and GPR41 (90, 91). In macrophages, butyrate activates GPR41 to down-regulate pro-inflammatory factors among which are nitric oxide synthase, tumor necrosis factor, interleukin 6, and monocyte chemoattractant protein-1 (92). In macrophages and neutrophils, SCFAs down-regulate interleukin 8 expression through activation of GPR43 and GPR41 (93). Finally, SCFAs can also regulate inflammation by activating anti-inflammatory signaling pathways by inhibiting histone deacetylase (86). Besides the role of SCFAs in inflammation, they also regulate the differentiation of T cells and B cells and regulate the function of innate immune cells among which are macrophages, neutrophils, and dendritic cells (86).

This study evaluated gut microbiome alterations in chronic pain patients compared to controls without chronic pain. Studies from different parts of the world were included among which were the USA, Europe, Asia, and Australia. There is no universal healthy gut microbiota (94, 95), since nationality and food preferences, among other factors, induce an influence on the gut microbiota. For example, the gut microbiome of a healthy European (including Slavic nationality) is characterized by the dominance of the phyla *Firmicutes*, *Bacteroidota*, *Actinobacteria, Proteobacteria, Fusobacteria*, and *Verrucomicrobia*, while the gut microbiome of Asians is very diverse and rich in members of the genera *Prevotella, Bacteroides Lactobacillus, Faecalibacterium, Ruminococcus, Subdoligranulum, Coprococcus, Collinsella, Megasphaera, Bifidobacterium*, and *Phascolarctobacterium* (96). Therefore, this study only included studies that compared gut microbiota to a control group to limit the influence of local differences in gut microbiota composition.

The field of chronic pain and gut microbiota composition is still in its infancy, wherefore condition-specific alterations remain to be elucidated when more research is available, in case the hypothesis of shared alterations is not valid in pain settings. The majority of studies explored chronic primary pain syndromes, wherefore gut dysbiosis in chronic secondary pain syndromes still needs to be explored in more detail. When interpreting the results of this study, it should be taken into account that medication was previously denoted as an important covariate, and more specifically antibiotics, osmotic laxatives, inflammatory bowel disease medication, female hormones, benzodiazepines, antidepressants, and antihistamines (60). Recently, a multi-omics analysis elaborated on the concept of opioid-induced dysbiosis in gut microbiota (97), which further supports the hypothesis of addressing the gut-brain axis in patients with chronic pain, especially in those patients who take opioids as pain medication. Medication use was reported for every individual study, however, it was not possible to take a numerical output for medication use into account in the conducted meta-analysis. As revealed by this review, there is no common pipeline to conduct laboratory analyses, statistical evaluations, or quality assurance for gut microbiome data. Future steps should be conducted towards harmonization of processing gut microbiome data to ensure better comparability of the results.

## 5 Conclusions

This review pointed towards the potential value of dysbiosis in chronic pain patients, with non-specific disease alterations of microbes.

## Data availability statement

The original contributions presented in the study are included in the article/**Supplementary Material**. Further inquiries can be directed to the corresponding author.

## Author contributions

LG: Conceptualization, Formal analysis, Methodology, Writing – original draft, Writing – review & editing. TD: Formal analysis, Writing – review & editing. JP: Investigation, Writing – review & editing. MB: Investigation, Writing – review & editing. MR: Investigation, Writing – review & editing. PR: Investigation, Writing – review & editing. MM: Conceptualization, Formal analysis, Investigation, Writing – review & editing.
