# Supplementary material for: Gut dysbiosis in patients with chronic pain: a systematic review and meta-analysis
Source: Front Immunol. 2024 Jan 30;15:1342833. doi: 10.3389/fimmu.2024.1342833 (PMC10862364; doi:10.3389/fimmu.2024.1342833)
Supplement: Supplementary file 1 [file DataSheet_1.docx]

**Supplementary Datasheet 1: search string in PubMed**

(((((((((((((chronic OR persistent OR prolonged OR intractable OR long-lasting OR "long lasting" OR long-term OR "long term" OR musculoskeletal OR widespread OR neuropathic OR cancer)) AND (pain)) OR ("complex regional pain syndrome")) OR (headache)) OR (neuralgia)) OR ("Chronic Pain"[Mesh])) OR ("Pain, Intractable"[Mesh])) OR ("Complex Regional Pain Syndromes"[Mesh])) OR ("Cancer Pain"[Mesh])) OR ("Headache"[Mesh])) OR ("Neuralgia"[Mesh])) OR ("Musculoskeletal Pain"[Mesh])) AND (((((((((gastrointestinal OR gut OR intestinal OR fecal OR feces OR faeces OR bacterial OR microbial)) AND ((microbiota OR microbiome OR flora OR dysbiosis))) OR ("gastrointestinal microbiome")) OR ("gut-brain axis")) OR ("gut brain axis")) OR ("Gastrointestinal Microbiome"[Mesh])) OR ("Microbiota"[Mesh])) OR ("Brain-Gut Axis"[Mesh]))
