# Supplementary material for: Gut dysbiosis in patients with chronic pain: a systematic review and meta-analysis
Source: Front Immunol. 2024 Jan 30;15:1342833. doi: 10.3389/fimmu.2024.1342833 (PMC10862364; doi:10.3389/fimmu.2024.1342833)
Supplement: Supplementary file 2 [file Table_1.docx]

**Supplementary Table 1: Risk of bias assessment**

| **Study** | **Selection1** | **Selection2** | **Selection3** | **Selection4** | **Comparability1** | **Exposure1** | **Exposure2** | **Exposure3** | **Total** | **Quality** |
| --- | --- | --- | --- | --- | --- | --- | --- | --- | --- | --- |
| Bai et al., 2022 | 0 | 1 | 1 | 0 | 0 | 1 | 1 | 0 | 4 | low |
| Berlinberg et al., 2021 | 1 | 0 | 1 | 0 | 0 | 1 | 1 | 1 | 5 | low |
| Braundmeier-Fleming et al., 2016 | 1 | 1 | 0 | 0 | 0 | 1 | 1 | 1 | 5 | low |
| Chen et al., 2020 | 0 | 0 | 0 | 0 | 2 | 1 | 1 | 1 | 5 | low |
| Clos-Garcia et al., 2019 | 1 | 1 | 0 | 1 | 2 | 1 | 1 | 1 | 8 | high |
| Frémont et al., 2013 | 0 | 0 | 0 | 0 | 0 | 1 | 1 | 1 | 3 | low |
| Giloteaux et al., 2016 | 1 | 0 | 1 | 0 | 0 | 1 | 1 | 1 | 5 | low |
| Guo et al., 2023 | 1 | 1 | 1 | 0 | 2 | 1 | 1 | 1 | 8 | high |
| Janulewicz et al., 2019 | 1 | 1 | 1 | 1 | 0 | 1 | 1 | 1 | 7 | moderate |
| Kitami et al., 2020 | 1 | 1 | 0 | 1 | 2 | 1 | 1 | 1 | 8 | high |
| Kopchak et al., 2022 | 0 | 0 | 0 | 0 | 0 | 1 | 1 | 0 | 2 | low |
| Lupo et al., 2021 | 0 | 0 | 0 | 1 | 2 | 1 | 1 | 1 | 6 | moderate |
| Mandarano et al., 2018 | 0 | 0 | 1 | 0 | 0 | 1 | 1 | 1 | 4 | low |
| Minerbi et al., 2019 | 1 | 1 | 1 | 0 | 0 | 1 | 1 | 1 | 6 | moderate |
| Nagy-Szakal et al., 2017 | 1 | 1 | 1 | 1 | 2 | 1 | 1 | 1 | 9 | high |
| Reichenberger et all., 2013 | 1 | 1 | 1 | 1 | 0 | 1 | 1 | 1 | 7 | moderate |
| Sheedy et al., 2009 | 1 | 0 | 0 | 0 | 0 | 1 | 1 | 1 | 4 | low |
| Shukla et al., 2015 | 1 | 1 | 0 | 1 | 2 | 1 | 1 | 1 | 8 | high |
| Weber et al., 2022 | 1 | 1 | 1 | 0 | 2 | 1 | 1 | 1 | 8 | high |
| Yong et al., 2023 | 1 | 1 | 1 | 1 | 2 | 1 | 1 | 0 | 8 | high |
| Zhao et al., 2021 | 1 | 1 | 0 | 0 | 0 | 1 | 1 | 1 | 5 | low |
