# Supplementary material for: Gut dysbiosis in patients with chronic pain: a systematic review and meta-analysis
Source: Front Immunol. 2024 Jan 30;15:1342833. doi: 10.3389/fimmu.2024.1342833 (PMC10862364; doi:10.3389/fimmu.2024.1342833)
Supplement: Supplementary file 3 [file Table_2.docx]

**Supplementary Table 2: Methodology of stool processing in included studies**

| **Author** | **Collection** | **Storage** | **DNA extraction method** | **Library preparation** | **Sequencing** | **Sequencing platform** | **Read ouput (mean)** | **Computational pipeline** | **Read Processing and quality control** | **Taxonomic Classiciation Tool** | **Similarity cutoff** | **Taxonomic reference database** |
| --- | --- | --- | --- | --- | --- | --- | --- | --- | --- | --- | --- | --- |
| Bai et al., 2022 | Swabs, inserted into collection tube with preservation solution, shipped ideally within 48h of collection. |  |  | Illumina MiSeq 515f-806r amplification protocol | 16S rRNA V4 | Illumina MiSeq 515f-806r amplification protocol |  | QIIME 2 |  | Trained classifiers based on Greengenes database | 99% | Greengenes |
| Berlinberg et al., 2021 | Rectal swabs, immediately on ice. | Frozen at -80°C until further use. | Qiagen AllPrep Power Fecal DNA/RNA kit | NEB Next Ultra II FS DNA Library Prep Kit | Shotgun metagenomics | Illumina NovaSeq6000 platform (San Diego, CA, USA) | 7.8 - 24.0 million reads per sample | HUMAnN 2.0 pipeline | Kneaddata 0.7.5, utilizing Trimmomatic v0.39 and Bowtie2 v2.3.5 | MetaPhlAn v2.0 |  | UniRef90  KEGG (Kyoto Encyclopedia of Genes and Genomes) |
| Braundmeier-Fleming et al., 2016 | At-home stool sample collection kit, after which samples were freezed and shipped within 48h to lab on wet ice. | Frozen at -80°C until further use. |  | Primers: 357F and 926R | 16S rRNA V3-V5 | Illumina MiSeq V2 | 50.000 reads per sample | QIIME and Galaxy |  | QIIME and Galaxy | 97% | Genbank |
| Chen et al., 2020 | At-home sample collection, after which samples were freezed and shipped within 48h to lab on dry ice. | Frozen at -80°C until further use. | PowerSoil kit (MoBio) |  | Paired-end metagenomic sequencing | Illumina HiSeq2000 |  | MOCAT pipeline |  | metaphlan2 |  | Kyoto Encyclopedia of Genes and Genomes (KEGG) databases (release 79.0) using BLASTP v2.26 |
| Clos-Garcia et al., 2019 | Sampling in hospital and stored at 4°C. | Frozen at -80°C until further use. | PSP Spin Stool DNA Plus kit (STRATEC Molecular®) |  | 16s rDNA V3-V4 | Illumina MiSeq sequencing system | 12.000 reads per sample | QIIME2 package (v. 2017.10) |  | GreenGenes 13_8 | 97% | GreenGenes 13_8 |
| Frémont et al., 2013 | Samples of homogenized stool were collected and directly mixed with 2 ml of Aquastool solution (Multi Target Pharmaceuticals, Salt Lake City, USA). | Frozen at -20°C within one hour, until further use. | DNeasy Blood and Tissue kit (Qiagen) | Primers: AGGATTAGATACCCTGGTA and CRRCACGAGCTGACGAC | 16S rRNA V5-V6 | 454 Life Sciences Genome Sequencer FLX instrument (Roche) | 6000-7000 reads per sample | | RDP classifier (v 2.1) with CE > 80% | | 80% |  |
| Giloteaux et al., 2016 | At-home collection and refrigerated in RNAlater (Life Technologies, Grand Island, NY), afterwards shipment. | Frozen at -80°C until further use. | PowerSoil-htp DNA isolation kit (MoBio Laboratories Ltd, Carlsbad, CA) | 515F and 806R primers | 16S rRNA V4 | Illumina MiSeq 2x250 bp platform | 98.093 reads per sample | QIIME 1.9.0 |  |  | 97% |  |
| Guo et al., 2023 | At-home collection kits 48 hours prior to study visit and refrigerated. Shipment to lab in insulated Styrofoam boxes with frozen and refrigerated gel packs. | Frozen at -80°C until further use. | modified protocol of the QIAmp DNA Stool Mini Kit (Qiagen Inc; Valencia CA, USA) | KAPA Hyper Prep kit (KK8504, Kapa Biosystems) | Shotgun metagenomics | Illumina HiSeq 4000 platform (Illumina, San Diego, CA, USA) | 27.8 million reads per sample | In-house | Cutadapt | Kraken2 |  | RefSeq |
| Janulewicz et al., 2019 | Second Genome’s stool collection vial with barcode and nucleic acid stabilizing solution. | Frozen at -20°C until further use. | Qiagen MagAttract PowerMicrobiome (Qiagen, Germantown, MD, USA) DNA/RNA Kit | PCR amplification with fusion primers designed for Illumina adapters and indexing barcodes. | 16S rDNA V4 | Illumina MiSeq (Illumina, Inc, San Diego, CA, USA) |  |  |  | Second Genome’s analysis software package |  |  |
| Kitami et al., 2020 | At-home collection, then storage at -20°C until transfer to lab. |  | Freeze-dried fecal samples were suspended in 10% sodium dodecyl sulfate, 10 mM Tris–HCl, and 1 mM EDTA(pH 8.0), then disrupted with 0.1-mm zirconia/silica beads (BioSpec Products) by shaking at 1500  rpm for 10 min. Afer centrifugation, bacterial DNA was purifed using 25:24:1 phenol–chloroform–isoamyl alcohol andprecipitated by ethanol and sodium acetat. | amplified using 27Fmod-338R primer pairs for 22 cycles, indexed using Nextera XT index primers | 16S rRNA V1-V2 | MiSeq (Illumina) |  | QIIME |  | RDP classifier | 97% | Greengenes Database |
| Kopchak et al., 2022 |  |  |  |  |  |  |  |  |  |  |  |  |
| Lupo et al., 2021 | In-hospital collection of fecal samples in sterile tubes. | Frozen at -80°C within one hour, until further use. | QIAmp DNA Stool Mini kit | Phusion Flash High-Fidelity Master Mix | 16S rRNA V3-V4 | MiSeq Illumina platform (Illumina Inc, San Diego, CA) | 6100 sequences per sample | Mothur v.1.39.5 | fastx-toolkit | NAST algorithm and a kmer approach | 97% | Greengenes database |
| Mandarano et al., 2018 | At-home collection and refrigerated in RNAlater. Overnight shipment. | Frozen at -80°C until further use. | QIAmp DNA Mini Kit (Qiagen, Valencia, CA, USA). | llumina_Euk_1391f universal forward primer and individually barcoded Illumina EukBr reverse primers, according to the Earth Microbiome protocol. | 18S rRNA V9 | Illumina MiSeq platform |  | Quantitative Insights into Microbial Ecology (QIIME) software (1.9.1) |  | BLAST | 97% | SILVA 119 database |
| Minerbi et al., 2019 | At-home using Omnigen Gut OM-200 kit (DNA Genotek, Ottawa, Ontario, Canada). Sample frozen at -20°C and provided to center within 10 days in dedicated thermal bag (Thermos, Schaumburg, IL) containing an ice pack, and monitored using an adhesive thermal indicator (Warm Mark; MesaLabs, Lakewood, CO), which was attached to the sample bag. | Frozen at -80°C until further use. | QIAamp PowerFecal DNA kit (Qiagen, Venlo, the Netherlands) | Primers: Forward: S-D-Bact-0785-a-S-18, GGMTTAGATACCCBDGTA Reverse: S-*-Univ-1100-a-A-15, GGGTYKCGCTCGTTR | 16S rRNA V5-V6 | MiSeq250 platform | 77.8046 reads per sample | ANCHOR pipeline | Mothur | BLASTn | >99% | NCBI curated bacterial and Archaea RefSeq, NCBI nr/nt, SILVA, Ribosomal Database Project |
| Nagy-Szakal et al., 2017 | At-home fecal samples 24 to 48 h prior to clinical visits, stored at −20 °C, and transferred to site in styrofoam boxes with ice packs. Shipped from site to labo on dry ice. | Frozen at -80°C until further use. | KAPA Hyper Prep kit (KK8504, Kapa Biosystems) |  | Shotgun metagenomic sequencing | llumina HiSeq 4000 platform (Illumina, San Diego, CA, USA) |  | Qiime (v1.8) | Pre-processed using prinseq (v0.20.3) for end trimming; low-quality and low-complexity reads filtered; adaptor sequences removed using cutadapt (v 1.8.3) | Metaphlan (v1.7.8), Humann2 (v0.7.1) |  |  |
| Reichenberger et all., 2013 | At-home fecal samples collection and placed in stool specimen contained under anaerobic conditions, stored in a coller at -4°C and transported at 4°C to the lab within 24h. | Frozen at -80°C. Analysis samples were shipped on dry ice for sequencing. | QIAamp DNA stool mini kit (Qiagen) | DNA capture beads were combined with a small sample of double-stranded DNA molecules/µl and amplified by emulsion PCR. | 16S rRNA V2 | Genome Sequencer FLX Titanium System (Roche, Nutley, New Jersey). | NS | Qiime (Quantitative Insights Into Microbial Ecology) | NS | RDP (Ribosomal Database Project) Classifier | 97% | Green genes |
| Sheedy et al., 2009 | Morning bowel motion was collected in a faecal container and immediately transported in a sealed anaerobic pouch system (Oxoid, Adelaide, Australia), by activating the Anaero Gen Compact (Oxoid, Adelaide, Australia) prior to the pouch being sealed. Samples were transported cold (<12˚C) to the laboratory and analyzed within 48h after collection. |  |  |  |  |  |  |  |  |  |  |  |
| Shukla et al., 2015 | At hospital collection in Protocult collection halts followed by self-transference to a 100 ml sterile capped contrainer and freezed at -20°C or on dry ice before transferred to long freezer. | Frozen at -80°C until further use. | QIAamp DNA Stool Mini Kit (Qiagen) | NS | 16S | 454 GS FLX+ (Roche, Branford, CT) | 2.160 reads per sample |  | NS | RDP Classifier |  |  |
| Weber et al., 2022 | At-home collection in stool collection tubes with DNA Stabilizer (Statec Molecular, Berlin, Germany). Sent to lab in cooled package with electronic thermometer for tracking a constant temperature (max T was 15°C for 24h). | Frozen at -80°C until further use. | Maxwell RSC Blood DNA Kit (Promega, Mannheim, Germany) | The variable V4 region was amplified using Mastermix 16s Complete PCR Kit (Molzym, Bremen, Germany) with primers 16s_515_fwd and 16s_806_rev. | 16S rRNA V4 region | Ion GeneStudio S5 System (Thermo Fisher Scientific, MA, USA) | 68.838 reads per sample | GALAXY using the QIIME2 2019.7 | NS | QIIME2 |  | SILVA rRNA database ver 132 |
| Yong et al., 2023 | At-home collection with SPL Korea, Seoul, Korea collection kit. Then frozen at -20°C for maximum 14 days. | Frozen at -70°C until further use. | FastDNA Spin Kit for Soil (MP Biomedicals, Irvine, California, USA) | Primers: 16S_V3_F and 16S_V4_R | 16S rRNA V3–V4 | Illumina MiSeq equipment (San Diego, California, USA). | 59.305 reads per sample | Chunlab program | NS | BLAST 2.2.22 |  | EzBioCloud database |
| Zhao et al., 2021 | Fresh fecal samples collected into sterile tubes and immediately freezed. | Frozen at -80°C until further use. | CTAB/SDS method | Sequencing libraries generated using TruSeq DNA PCR-Free Sample Preparation Kit (Illumina) | 16S rRNA V3-V4 | Illumina HiSeq 2500 PE250 platform | 78.054 reads per sample | QIIME (V1.9.1) | NS | Mothur algorithm | 97% | GreenGenes 13_8 |
